# Inhibition of *Henipavirus *fusion and infection by heptad-derived peptides of the *Nipah virus *fusion glycoprotein

**DOI:** 10.1186/1743-422X-2-57

**Published:** 2005-07-18

**Authors:** Katharine N Bossart, Bruce A Mungall, Gary Crameri, Lin-Fa Wang, Bryan T Eaton, Christopher C Broder

**Affiliations:** 1 CSIRO Livestock Industries, Australian Animal Health Laboratory, Geelong, Victoria 3220, Australia; 2 Department of Microbiology and Immunology, Uniformed Services University, Bethesda, MD 20814, USA

**Keywords:** Paramyxovirus, Hendra virus, Nipah virus, envelope glycoprotein, fusion, infection, inhibition, antiviral therapies

## Abstract

**Background:**

The recent emergence of four new members of the paramyxovirus family has heightened the awareness of and re-energized research on new and emerging diseases. In particular, the high mortality and person to person transmission associated with the most recent Nipah virus outbreaks, as well as the very recent re-emergence of Hendra virus, has confirmed the importance of developing effective therapeutic interventions. We have previously shown that peptides corresponding to the C-terminal heptad repeat (HR-2) of the fusion envelope glycoprotein of Hendra virus and Nipah virus were potent inhibitors of both Hendra virus and Nipah virus-mediated membrane fusion using recombinant expression systems. In the current study, we have developed shorter, second generation HR-2 peptides which include a capped peptide via amidation and acetylation and two poly(ethylene glycol)-linked (PEGylated) peptides, one with the PEG moity at the C-terminus and the other at the N-terminus. Here, we have evaluated these peptides as well as the corresponding scrambled peptide controls in Nipah virus and Hendra virus-mediated membrane fusion and against infection by live virus *in vitro*.

**Results:**

Unlike their predecessors, the second generation HR-2 peptides exhibited high solubility and improved synthesis yields. Importantly, both Nipah virus and Hendra virus-mediated fusion as well as live virus infection were potently inhibited by both capped and PEGylated peptides with IC_50 _concentrations similar to the original HR-2 peptides, whereas the scrambled modified peptides had no inhibitory effect. These data also indicate that these chemical modifications did not alter the functional properties of the peptides as inhibitors.

**Conclusion:**

Nipah virus and Hendra virus infection *in vitro *can be potently blocked by specific HR-2 peptides. The improved synthesis and solubility characteristics of the second generation HR-2 peptides will facilitate peptide synthesis for pre-clinical trial application in an animal model of *Henipavirus *infection. The applied chemical modifications are also predicted to increase the serum half-life *in vivo *and should increase the chance of success in the development of an effective antiviral therapy.

## Background

Two novel zoonotic paramyxoviruses have emerged to cause disease in the past decade, Hendra virus (HeV) in Australia in 1994–5 [1], and Nipah virus (NiV) in Malaysia in 1999 [2]. HeV and NiV caused severe respiratory and encephalitic disease in animals and humans (reviewed in [3,4]), HeV was transmitted to humans by close contact with infected horses; NiV was passed from infected pigs to humans. Both are unusual among the paramyxoviruses in their ability to infect and cause potentially fatal disease in a number of host species, including humans. Both viruses also have an exceptionally large genome and are genetically closely related yet distinct from all other paramyxovirus family members. Due to their unique genetic and biological properties, HeV and NiV have been classified as prototypic members of the new genus *Henipavirus*, in the family Paramyxoviridae [5,6]. Serological surveillance and virus isolation studies indicated that HeV and NiV reside naturally in flying foxes in the genus *Pteropus *(reviewed in [7]). Investigation of possible mechanisms precipitating their emergence indicates ecological changes resulting from deforestation, human encroachment into bat habitats and high intensity livestock farming practices as the likely primary factors [7]. Because these viruses are harboured in a mammalian reservoir whose range is vast, both HeV and NiV have the capability to cause disease over a large area and in new regions where disease was not seen previously. There have been several other suspected NiV occurrences since its recognition in 1999. Recently two confirmed outbreaks in 2004 in Bangladesh caused fatal encephalitis in humans and for the first time, person-to-person transmission appeared to have been a primary mode of spread [8-12]. In addition, there appeared to be direct transmission of the virus from the flying fox to humans, and the case mortality rate was ~70%; significantly higher than any other NiV outbreak to date. Currently, HeV and NiV are categorized as biological safety level-4 (BSL-4) pathogens, and NiV has also been classified as a category C priority pathogen. Category C agents include emerging pathogens that could be engineered for mass dissemination in the future because of availability; ease of production and dissemination; and potential for high morbidity and mortality and major health impact. All of the above reasons illustrate why an effective antiviral therapy is needed for henipaviruses.

Paramyxoviruses contain two membrane-anchored glycoproteins that are required for virion attachment to and fusion with the membrane of the host cell. Depending on the biological properties of the virus, the attachment protein is referred to as either the hemagglutinin-neuraminidase (HN), the hemagglutinin (H), or the G glycoprotein which lacks hemagglutinating and neuraminidase activities. Whereas most paramyxoviruses employ sialic acid moieties as receptors, HeV and NiV make use of a cell-surface expressed protein and their G glycoprotein binds to ephrin-B2 on host cells [13]. The fusion protein (F) facilitates the fusion of virion and host cell membranes during virus infection, and is an oligomeric homotrimer [14,15]. The biologically active F protein consists of two disulfide linked subunits, F_1 _and F_2_, which are generated by the proteolytic cleavage of a precursor polypeptide known as F_0 _(reviewed in [16,17]). In all cases the membrane-anchored subunit, F_1_, contains a new amino terminus that is hydrophobic and highly conserved across virus families and referred to as the fusion peptide (reviewed in [18]). There have been considerable advances in the understanding of the structural features and development of mechanistic models of how several viral envelope glycoproteins function in driving the membrane fusion reaction (reviewed in [19-21]). One important feature of many of these fusion glycoproteins are two α-helical domains referred to as heptad repeats (HR) that are involved in the formation of a trimer-of-hairpins structure [22,23]. HR-1 is located proximal to the amino (N)-terminal fusion peptide and HR-2 precedes the transmembrane domain near the carboxyl (C)-terminus [22,24-26]. For many viral fusion glycoproteins the N-terminal HR-1 forms an interior, trimeric coiled-coil surrounded by three anti-parallel helices formed from HR-2 (reviewed in [18]). Both the HeV and NiV F glycoprotein HR domains have been shown to interact with each other and form the typical 6-helix coiled-coil bundles [24,27].

Peptide sequences from either HR domain of the F glycoprotein of several paramyxoviruses, including HeV and NiV have been shown to be inhibitors of fusion [25,28-35]. Targeting this membrane fusion step of the viral infection process has garnered much attention, primarily lead by work on human immunodeficiency virus type 1 (HIV-1) (reviewed in [36]). Indeed, the HIV-1 envelope derived peptide, enfuvirtide (Fuzeon™, formerly T-20), has been clinically successful [37,38]. Enfuvirtide is a 36-amino acid peptide corresponding to a portion of the C-terminal HR-2 domain of the gp41 subunit of the envelope glycoprotein. Approved by the FDA in March 2003, enfuvirtide has been shown to be comparable to other anti-retroviral therapeutics in terms of reducing viral load, and is generally well tolerated despite its parenteral administration, and enfuvirtide has added significantly to optimized combination therapy in a growing number of patients with multiple HIV-1 resistance to the currently available antiretroviral drugs [39].

No therapeutic treatments are currently available for HeV or NiV infection. In our previous studies, we demonstrated that peptides derived from the HR-2 of either the HeV or NiV F were potent inhibitors of fusion [34]. However, although these peptides were effective, their specific properties such as overall length where not optimized, and they were large and somewhat insoluble making synthesis and purification problematic. In preparation to evaluate these peptides as potential therapeutic fusion inhibitors against NiV and HeV infection, second generation versions were designed with changes aimed at improving their solubility and *in vivo *half-life when administered to animals. In the current study, we have produced shorter 36 amino acid capped peptides by amidation at the N-terminus and acetylation at the carboxyl-terminus. In addition, two alternate peptide versions were made with the addition of a poly(ethylene glycol) moiety to either the C-terminus or the N-terminus. Here we report on the biological activity of these modified peptides and demonstrate that chemical modification increased solubility significantly without altering their biological properties of inhibiting membrane fusion. Further, all three versions were capable of blocking both fusion as well as live HeV and NiV infection with IC_50 _concentrations in the nM range, similar to those reported with other viral systems.

## Results

### Heptad peptide inhibition of Hendra virus and Nipah virus-mediated cell-cell fusion

**Figure 1:**
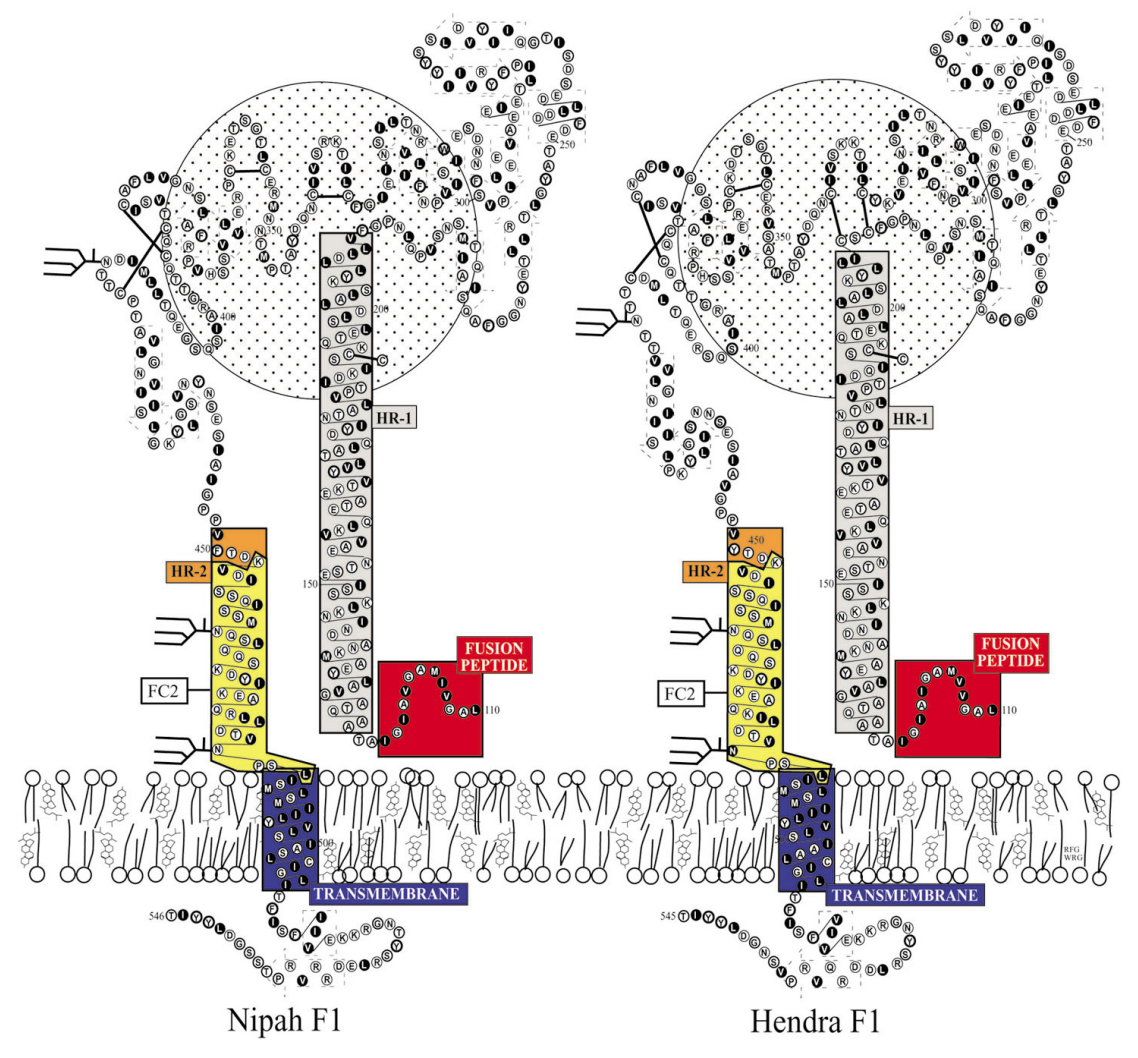
**Hypothetical models of the transmembrane (F1) glycoproteins of Hendra virus and Nipah virus**. The models are derived by homology modeling with the known structure of the F protein of Newcastle disease virus [40]. These models are consistent protein structures predicted by the computer algorithms PHDsec [41] and TMpred [42] as described in the Methods. The heptad repeats are indicated as HR-1 (grey) and HR-2 (yellow/orange), transmembrane anchor (blue). The F_2 _subunit is represented by the circle behind the F_1 _subunit. The 36 amino acid fusion inhibitor peptide sequence used in the present study is designated as FC2 and is boxed (yellow). The equivalent location of FC2 in the HeV F1 subunit is shown for comparison.

Hypothetical models of the transmembrane (F1) glycoproteins of HeV and NiV are shown in Fig. 1. The models are derived by homology modeling with the known structure of the F protein of Newcastle disease virus [40]. These models are consistent protein structures predicted by the computer algorithms PHDsec [41] and TMpred [42]. Overall, the structures of the HeV and NiV F_1 _transmembrane subunit, including the heptad repeats (HR-1 and HR-2 helices), closely resemble that of the gp41 subunit of the HIV-1 envelope glycoprotein [43-45]. The depicted circle in the background represents the F_2 _subunit of NiV F. Due to the structural similarities and clinical success of the gp41 heptad peptides, we hypothesized that peptides derived from the HR-2 of HeV or NiV F would be effective antiviral therapies for henipavirus infection. In previous studies we evaluated the inhibition properties of 42 amino acid length peptides derived from both the N and C-terminal heptad repeats (HR-1 and HR-2) of HeV and NiV F in a vaccinia virus-based reporter gene assay that quantitatively measured cell-cell fusion mediated by the envelope glycoproteins of HeV and NiV [25,34]. Although both HR-1 and HR-2 derived peptides exhibited fusion inhibitory activity, the HR-2 peptide (residues 447–489) was more potent and more soluble. The HeV and NiV HR-2 peptides differed at three locations (amino acids 450, 479 and 480) with phenylalanine, arginine and leucine in NiV replaced by tyrosine, lysine and isoleucine in HeV [6,46]. These differences in the sequence of either peptide did not alter their homologous or heterologous inhibitory activity, suggesting that either peptide possessed potential therapeutic activity to both HeV and NiV. Here, we designed second generation versions of the NiV based HR-2 derived peptide with changes aimed at improving their solubility and *in vivo *half-life when administered to animals. Shorter, 36 amino acid capped peptides were synthesized (sequence denoted as FC2 in Fig. 1) by amidation at the N-terminus and acetylation at the carboxyl-terminus, modifications known to have improved *in vivo *half-life of Fuzeon™ (Thomas Matthews, Trimeris Inc., personal communication). In addition, two alternate peptide versions were made with the addition of a poly(ethylene glycol) moiety to either the C-terminus or the N-terminus which improved peptide solubility during preparation, and may also potentially improve the pharmacokinetics *in vivo *[47,48].

**Figure 2:**
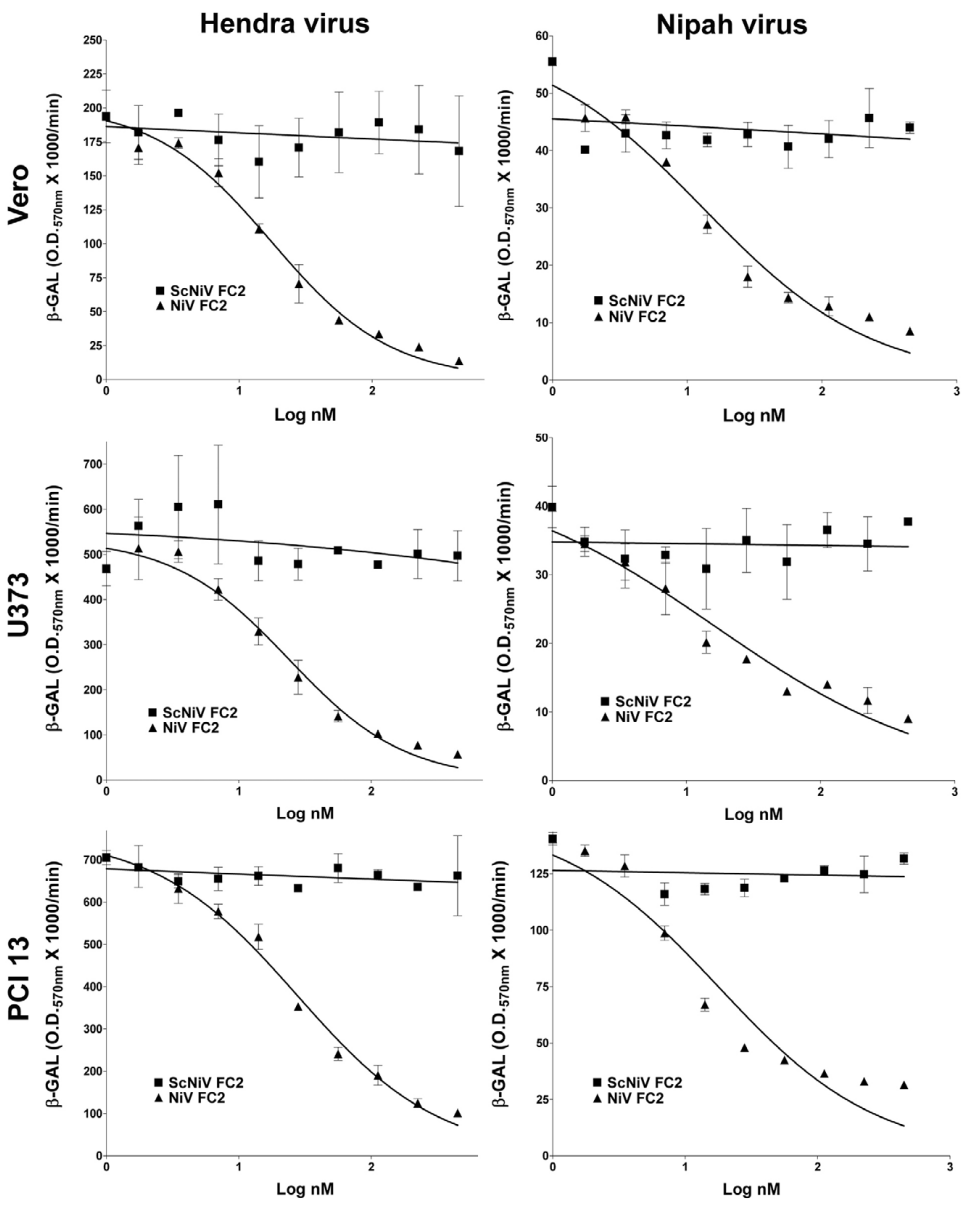
**Inhibition of Hendra virus and Nipah virus-mediated cell-cell fusion by capped C-terminal heptad peptide NiV FC2**. HeLa cells were infected with vaccinia recombinants encoding HeV F and HeV G or NiV F and NiV G glycoproteins, along with a vaccinia recombinant encoding T7 RNA polymerase (effector cells). Each designated target cell type was infected with the *E. coli *LacZ-encoding reporter vaccinia virus vCB21R. Each target cell type (1 × 10^5^) was plated in duplicate wells of a 96-well plate. Inhibition was carried out using either capped NiV FC2 or ScNiV FC2 (control) heptad peptide. Peptides were added to the HeV or NiV glycoprotein-expressing cells (1 × 10^5^), incubated for 30 min at 37°C, and then each target cell type was added. The cell fusion assay was performed for 2.5 hr at 37°C, followed by lysis in Nonidet P-40 (1%) and β-Gal activity was quantified.

First, we examined the activity of the capped peptides on HeV and NiV-mediated membrane fusion. In previous studies, un-capped heptad-derived peptides had to be dissolved initially in 100% DMSO at concentrations between 50 and 500 μg/ml and then diluted in medium in order to maintain solubility. Here, the capped heptad-derived peptide (capped-NiV FC2) was completely soluble and dissolved in cell culture medium at concentrations as high as 10 mg/ml. For cell-cell fusion, envelope expressing-effector cells were added to peptides prior to the addition of target cells. Shown in Fig. 2 are the dose-dependent inhibition profiles of HeV (column one) and NiV-mediated (column 2) cell-cell fusion mediated by the capped-NiV FC2 peptide in Vero (Fig. 2A), U373 (Fig. 2B), and PCI 13 (Fig. 2C) cell lines. The scrambled, capped, control peptide (capped-ScNiV FC2) had no inhibitory effect, over the same concentration range, on the cell-fusion mediated by either virus in any of the three cell lines. NiV-mediated fusion appeared to be slightly more sensitive to peptide inhibition in comparison to the cell-fusion activity of HeV, although the calculated IC_50 _concentrations in each were comparable (Table 1). Importantly, the IC_50 _values of the capped version of NiV FC2 in these *in vitro *cell-cell fusion assays were within the 13–27 nM range, similar to what was observed in prior studies utilizing un-capped versions of the 42 amino acid heptad-derived peptides which yielded IC_50 _values between 5.2 and 5.8 nM [34].

**Table 1:** Summary of 50% inhibitory concentration values of peptide fusion inhibitors in cell-cell fusion and virus infection assays.

	Virus	Cell line	IC_50_* Capped NiV FC2 (nM)	IC_50 _N-PEG NiV FC2 (nM)	IC_50 _C-PEG NiV FC2 (nM)
Fusion Inhibition	HeV	Vero	17.59	6.54	142.4
	NiV	Vero	13.08	3.66	98.05
	HeV	U373	23.91	9.71	78.07
	NiV	U373	16.28	4.85	79.19
	HeV	PCI 13	27.54	6.18	147.2
	NiV	PCI 13	17.79	5.04	93.32

Live virus Inhibition	HeV	Vero	4.17	0.46	14.28
	NiV	Vero	11.42	1.36	43.76
	HeV	PCI 13	53.51	2.05	11.94
	NiV	PCI 13	2.70	1.26	55.57

*All IC_50_s were calculated using the non-linear regression function of GraphPad Prism software.

**Figure 3:**
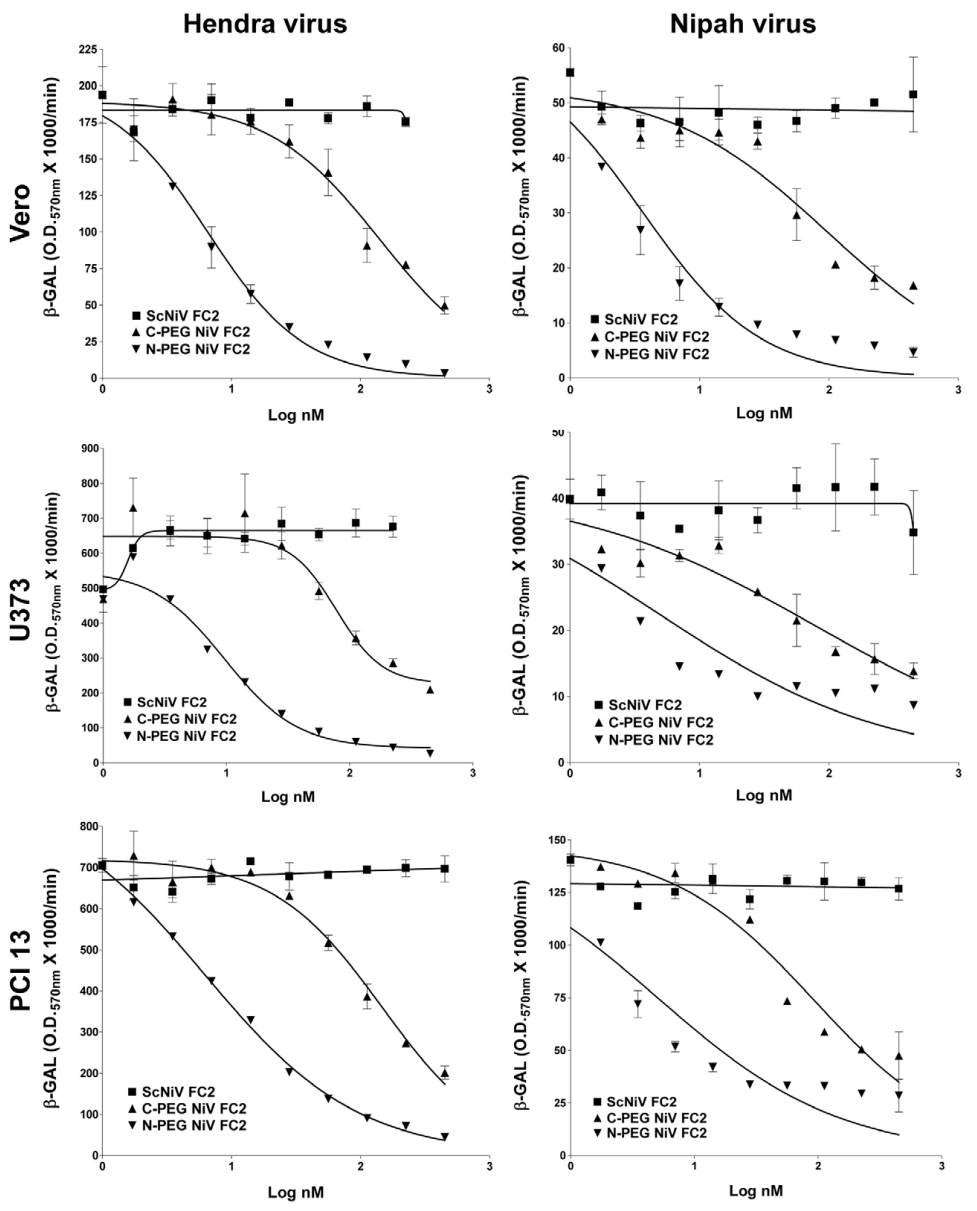
**Inhibition of Hendra virus and Nipah virus-mediated cell-cell fusion by N-terminal and C-terminal (PEG_10_) pegylated heptad peptide NiV FC2**. HeLa cells were infected with vaccinia recombinants encoding HeV F and HeV G or NiV F and NiV G glycoproteins, along with a vaccinia recombinant encoding T7 RNA polymerase (effector cells). Each designated target cell type was infected with the *E. coli *LacZ-encoding reporter vaccinia virus vCB21R. Each target cell type (1 × 10^5^) was plated in duplicate wells of a 96-well plate. Inhibition was carried out using either the N-terminal (N-PEG-NiV FC2) or C-terminal (C-PEG-NiV FC2) pegylated and capped heptad peptides or C-terminal pegylated scrambled control peptide (C-PEG-ScNiV FC2). Peptides were added to the HeV or NiV glycoprotein-expressing cells (1 × 10^5^), incubated for 30 min at 37°C, and then each target cell type was added The cell fusion assay was performed for 2.5 hr at 37°C, followed by lysis in Nonidet P-40 (1%) and β-Gal activity was quantified.

Using the cell-cell fusion assay we next examined the PEG-modified versions of NiV FC2. As predicted, these pegylated heptad peptides also possessed increased solubility characteristics and could be readily prepared at concentrations up to 10 mg/ml. The dose-response inhibition results of the N-PEG-NiV FC2 and C-PEG-NiV FC2 peptides are shown in Fig. 3, and inhibition was demonstrated in Vero (Fig. 3A), U373 (Fig. 3B), and PCI 13 (Fig. 3C) cell lines. Both pegylated versions of NiV FC2 were capable of blocking NiV and HeV-mediated cell-fusion, while the scrambled PEG-control peptide (C-PEG-ScNiV FC2) had no inhibitory activity. Because of the required specificity of the heptad peptide amino acid sequence to convey fusion inhibitory activity, as well as the high cost of peptide synthesis, we chose to only synthesize one version of the scrambled peptide as a pegylated control with the PEG_10 _moiety linked to the C-terminus. It was also noted that the NiV FC2 peptide with the PEG_10 _moiety added to the C-terminus had significantly reduced inhibitory capacity, as compared to PEG_10 _added to the N-terminus, against both NiV and HeV-mediated cell-fusion in all three cell lines tested. The reduction of C-PEG-NiV FC2 activity versus N-PEG-NiV FC2 was approximately 20-fold in all cases (Table 1) with the exception of HeV-mediated cell-fusion with the U373 cell line (Fig. 3B). Importantly, in all cases, the N-PEG-NiV FC2 demonstrated very similar IC_50_s (3–10 nM) to what was observed in prior studies utilizing un-capped versions of the 42 amino acid heptad-derived peptides (5–6 nM).

### Heptad peptide inhibition of Hendra virus and Nipah virus infection

**Figure 4:**
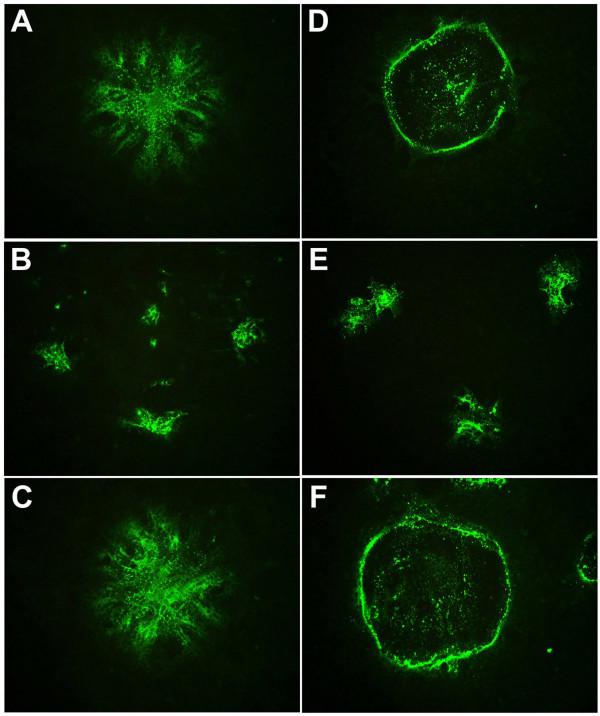
**Immunofluorescence-based syncytia assay of Hendra virus and Nipah virus infection**. Vero cells were plated into 96 well plates and grown to 90% confluence. Cells were pre-treated with heptad peptides for 30 min at 37°C prior to infection with 1.5 × 10^3 ^TCID_50_/ml and 7.5 × 10^2 ^TCID_50_/ml of live HeV or NiV (combined with peptide). Cells were incubated for 24 hours, fixed in methanol and immunofluorescently stained for P protein prior to digital microscopy. Images were obtained using an Olympus IX71 inverted microscope coupled to an Olympus DP70 high resolution color camera and all images were obtained at an original magnification of 85×. Representative images of FITC immunofluorescence of anti-P labeled HeV and NiV syncytia are shown. A: HeV without peptide. B: HeV with C-PEG-NiV FC2. C: HeV with N-PEG-ScNiV FC2. D: NiV without peptide. E: NiV with N-PEG-NiV FC2. F: NiV with N-PEG-ScNiV FC2.

We next sought to confirm the inhibitory activity of Nipah virus heptad-derived peptides on the infection of live HeV and NiV in cell culture. We routinely employ Vero cell culture to perform live henipavirus infection assays, as well as in the propagation of virus stocks. The infection of Vero cells with HeV or NiV produced characteristic syncytial morphologies for each virus [49]. HeV reproducibly incorporated surrounding cells in the culture monolayer into each syncytium with the cell nuclei and viral proteins spread throughout the majority of the giant cell. In contrast, NiV infected syncytia initially demonstrated a similar appearance to their HeV counterparts, but characteristically both cell nuclei and viral protein were later sequestered around the periphery of each giant cell leaving the central region largely empty. In order to assess the extent of viral infection, we have developed an assay that will detect viral protein by immunofluorescence staining and localization of the P protein using a cross-reactive anti-P peptide-specific antiserum. Using this syncytia-based immunofluorescence infection assay, we initially tested the N-PEG NiV FC2 peptide for its ability to block virus infection and results are shown in Fig. 4. In the absence of peptide, the different syncytial morphologies of HeV and NiV- infected cells were clearly evident. In the HeV-infected syncytia (Fig. 4A), the viral P protein was spread throughout the majority of the giant cell; whereas, the NiV-infected syncytia (Fig. 4D) were circular structures delineated by a ring of the viral antigen. Incubation of 500 nM N-PEG-NiV FC2 with either HeV (Fig. 4B) or NiV (Fig. 4E) infected cells resulted in a dramatic and robust reduction in syncytial size although the number of syncytia per cell monolayer remained largely unchanged. In parallel, the incubation of 500 nM C-PEG-ScNiV FC2 control peptide with HeV or NiV-infected cells (Fig. 4C and 4F respectively) revealed a syncytial morphology and size identical to those observed in the absence of any peptide.

**Figure 5:**
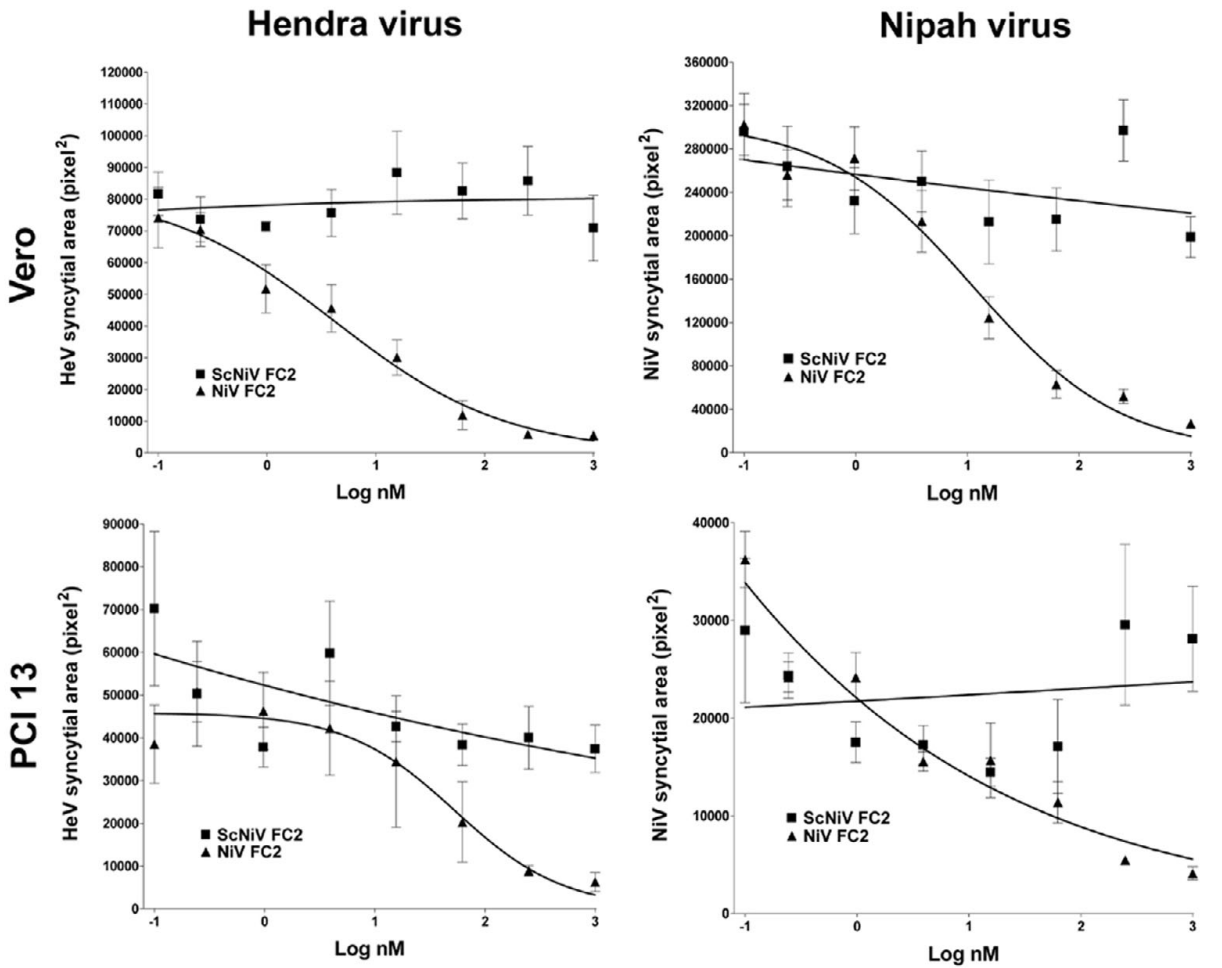
**Inhibition of Hendra virus and Nipah virus infection by capped heptad peptides**. Vero cells or PCI 13 cells were plated into 96 well plates and grown to 90% confluence. Cells were pre-treated with the indicated peptide for 30 min at 37°C prior to infection with 1.5 × 10^3 ^TCID_50_/ml and 7.5 × 10^2 ^TCID_50_/ml of live HeV or NiV (combined with peptide). Cells were incubated for 24 hours, fixed in methanol and immunofluorescently labeled for P protein prior to digital microscopy and image analysis to determine the relative area of each syncytium (see Methods). The figure shows the relative syncytial area (pixel^2^) versus the indicated peptide concentration for HeV and NiV.

**Figure 6:**
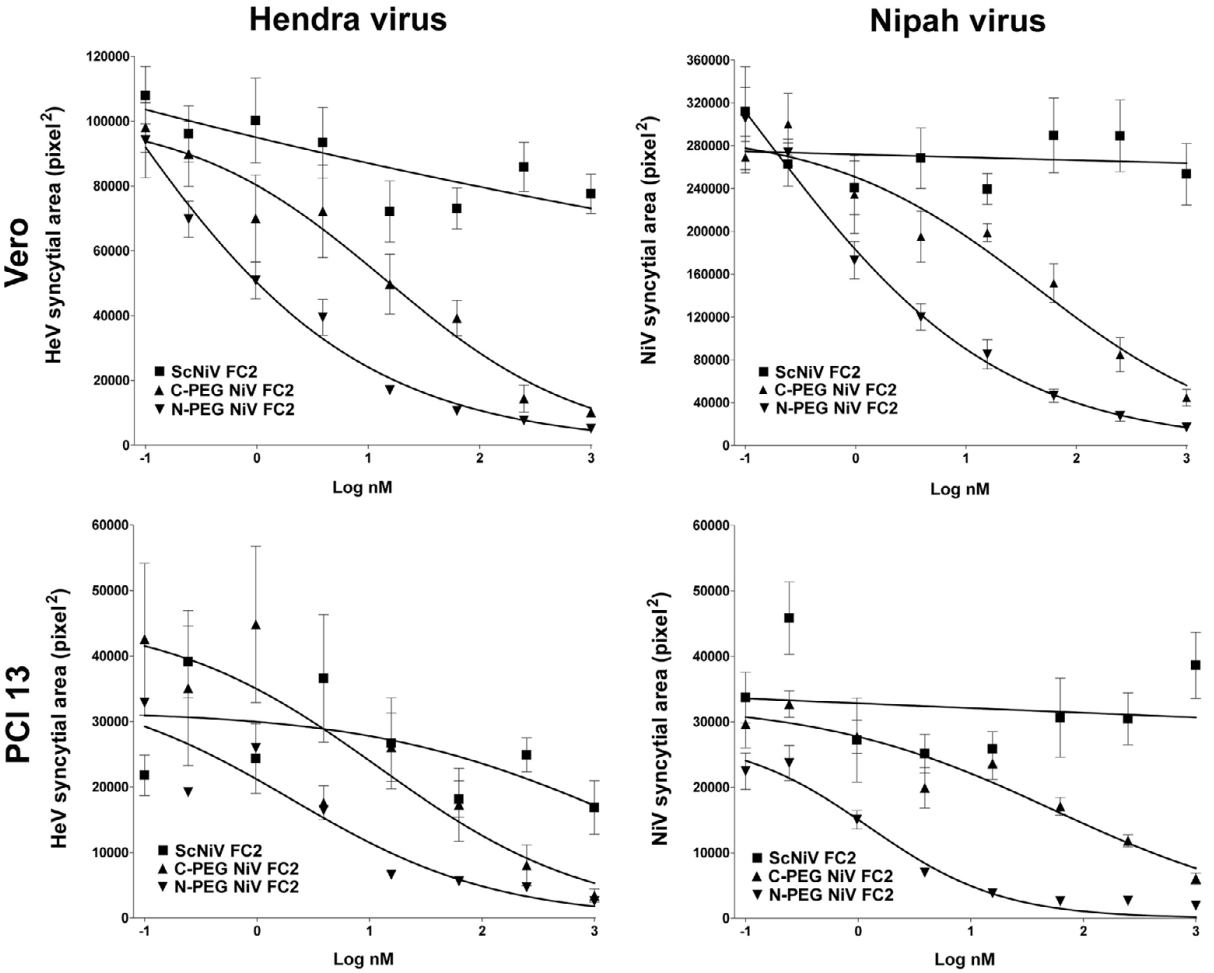
**Inhibition of Hendra virus and Nipah virus infection by N-terminal and C-terminal pegylated heptad peptides**. Vero cells or PCI 13 cells were plated into 96 well plates and grown to 90% confluence. Cells were pre-treated with the indicated peptide for 30 min at 37°C prior to infection with 1.5 × 10^3 ^TCID_50_/ml and 7.5 × 10^2 ^TCID_50_/ml of live HeV or NiV (combined with peptide). Cells were incubated for 24 hours, fixed in methanol and immunofluorescently labeled for P protein prior to digital microscopy and image analysis to determine the relative area of each syncytium (see Methods). The figure shows the relative syncytial area (pixel^2^) versus the indicated peptide concentration for HeV and NiV.

We next used the syncytia-based immunofluorescence infection assay to examine all of the peptides over a range of concentrations in two different cell lines. We further preformed a quantitative analysis of syncytial areas based on immunofluorescence detection of viral antigen for HeV and NiV (see Materials and Methods) and revealed a grading of syncytial area inversely proportional to peptide concentration. Shown in Fig. 5 is the quantitative analysis of the syncytial area observed in HeV and NiV infection of both Vero (Fig. 5A and 5B) and PCI 13 (Fig. 5C and 5D) cell cultures over a range of concentrations of the capped-NiV FC2 peptide. In all cases significant inhibition of HeV and NiV infection and spread is observed in comparison to the scrambled capped control peptide (capped-ScNiV FC2). Similarly, shown in Fig. 6, both the N-PEG and C-PEG NiV FC2 peptides possessed potent inhibitory activity on HeV and NiV infection in Vero (Fig. 6A and 6B) and PCI 13 (Fig. 6C and 6D) cell cultures. Again, the scrambled C-PEG control peptide (C-PEG-ScNiV FC2) had no effect at any concentration tested. As was observed in the cell-cell fusion assays, in all cases, the C-PEG-NiV FC2 peptide exhibited weaker inhibitory activity in blocking virus infection, spread and syncytial size in comparison to the N-PEG-NiV FC2. The N-PEG-NiV FC2 peptide had considerable potency against both NiV and HeV and the calculated IC_50 _values for inhibiting either virus on both cell lines ranged from 0.46 nM to 2.05 nM (Table 1).

## Discussion

Both NiV and HeV continue to re-emerge, and in early 2004 two NiV outbreaks in Bangladesh have been confirmed totalling some 53 human cases of infection, and HeV has reappeared in Northern Australia in late 2004 with two cases of fatal infection in horses and one non-fatal human case [50]. The most recent NiV occurrence has again appeared in Bangladesh in January of 2005 [51]. Several important observations in these most recent outbreaks of NiV have been made, including a higher incidence of acute respiratory distress syndrome, person-to-person transmission occurring in the majority of cases, and significantly higher case fatality rates (60–75%), and no direct link to infected livestock or domestic animals [8-12,51]. In particular, the availability of NiV in the environment and the ability to grow the virus to high titer in the laboratory, it is also now considered a potential biological terror agent. Taken together these observations highlight the need to explore therapeutic strategies for henipaviruses. While there is some evidence that ribavirin therapy may be of clinical benefit [52], there are currently no other specific treatment options and only supportive care is indicated.

Paramyxoviruses, like retroviruses, possess a class I membrane fusion mechanism, and there have been major recent advances in the understanding of the structural requirements and mechanisms involved in the fusion process mediated by these viruses (reviewed in [19,53-55]). The present model of class I membrane fusion describes the formation of a trimer-of-hairpins structure whose oligomeric coiled-coil formation is mediated by the 2 α-helical heptad repeat domains of the fusion glycoprotein which drives membrane fusion. Peptides corresponding to either of these heptad domains block fusion by interfering with the formation of the trimer-of-hairpins structure, first noted with sequences derived from the gp41 subunit of the HIV-1 envelope glycoprotein [56,57]. HIV-1 heptad-peptides have now met with clinical success and are the first approved fusion inhibitor therapeutics for a viral infection. Peptide sequences from either the N or C heptads of the F glycoprotein from a variety of paramyxoviruses have also been shown to inhibit fusion [28-33,58]. Previously, we demonstrated that fusion-inhibiting peptides corresponding to the C-terminal heptad repeat domain of the F glycoprotein of either HeV or NiV could potently inhibit the membrane fusion activity of either virus [25,34]. Because the peptides derived from the HR-2 of NiV F could inhibit both HeV and NiV-mediated fusion, in this study we only pursued peptides derived from NiV F. Furthermore, we have refined our initial peptide fusion inhibitors by reducing their length and chemically modifying their amino and carboxyl termini either by amidation or acetylation or through the addition of a PEG_10 _moiety, and have examined these new peptides in both membrane fusion and virus infection assays.

We have demonstrated that *Henipavirus*-mediated fusion and infection can be potently inhibited by these chemically modified peptides *in vitro *in a dose-dependent fashion. Overall, the IC_50 _concentrations of the peptides in the present study were similar to our previous observations on un-capped 42-mer peptides against HeV and NiV-mediated cell-cell fusion as well as to those observed in other paramyxovirus and retrovirus systems. However, we found that the N-terminal pegylated NiV FC2 peptide used here to be particularly potent with overall IC_50 _values of <10 nM for both HeV and NiV cell-cell fusion and virus infection. The present results indicate that both the capped and pegylated peptides are equally as effective as the unmodified first generation fusion-inhibiting peptides. Interestingly, peptides with the PEG_10 _moiety linked to the C-terminus were slightly, yet reproducibly, less effective than N-terminal pegylated peptides. We speculate that this could reflect some process of steric hindrance effect by the PEG_10 _moiety in interacting with the F glycoprotein during its conformational alteration leading to 6-helix bundle formation.

These same chemical modifications also improved the solubility characteristics of the heptad-derived peptides, and also significantly increased the yield during synthesis and purification (data not shown). The primary objectives of the present study were to demonstrate that these peptides possessed potent inhibitory activity in surrogate viral glycoprotein-mediated membrane fusion assays as well as in live virus infection assays, and improve peptide solubility and synthesis yields. The specific chemical modifications were chosen, especially pegylation, to help improve the plasma half-life and thus enhance therapeutic success. Covalent coupling of PEG to proteins or "pegylation" is currently considered one of the most successful techniques to prolong the residence time of protein drugs in the bloodstream [47,59-61]. PEG is a water soluble polymer that when covalently linked to molecules, conveys its physico-chemical properties and therefore modifies the biodistribution and solubility of peptide and non-peptide drugs. Additionally, pegylation masks the peptide's surface and increases the molecular size of the polypeptide, thus reducing its renal ultrafiltration. PEG modification can also prevent the approach of antibodies or antigen processing cells and reduce their degradation by proteolytic enzymes [62].

## Conclusion

The isolation of four new members of the family *Paramyxoviridae *in the past 10 years in addition to several largely uncharacterised paramyxoviruses recovered from historical rodent and snake sampling may indicate a much larger than previously thought, reservoir of paramyxoviruses. Given the success of heptad-derived peptide inhibition of paramyxovirus fusion, many of these new and yet to be discovered viruses may well be inherently treatable, such that the possibility of antiviral therapy may be available as soon as a sequence has been obtained for the respective fusion envelope glycoprotein. We are presently evaluating the properties of these anti-HeV and NiV fusion inhibiting peptides as well as their potential therapeutic value with an *in vivo *model of virus infection.

## Methods

### Cells and Culture conditions

HeLa cells (ATCC CCL 2) and African green monkey (Vero) cells (ATCC CCL 81) were obtained from the American Type Culture Collection. A HeLa cell line derivative (HeLa-USU) which does not express the NiV and HeV receptor, ephrin-B2 [13] was provided by Anthony Maurelli, USUHS, Bethesda, MD. The human glioblastoma cell line U373-MG was provided by Adam P. Geballe, Fred Hutchinson Cancer Research Center, Seattle, WA [63]. The human head and neck carcinoma PCI 13 cell line was the kind gift of Ernest Smith, Vaccinex, Inc. HeLa and U373 cell monolayers were maintained in Dulbecco's modified Eagle's medium supplemented with 10% cosmic calf serum (CCS) (Hyclone, Logan, UT) and 2 mM L-glutamine (DMEM-10). PCI 13 cell monolayers were maintained in DMEM-10 supplemented with 1 mM HEPES. Vero cells were maintained in the absence of antibiotics in Minimal Essential Medium containing Earle's salts and 10% fetal calf serum (EMEM-10). All cell cultures were maintained at 37°C under a humidified 5% CO2 atmosphere.

### Viruses

For expression of recombinant HeV and NiV F and G glycoproteins, the following recombinant vaccinia viruses were employed: vKB7 (NiV F), vKB6 (NiV G), vKB1 (HeV F), vKB2 (HeV G) [25,34,64]. Bacteriophage T7 RNA polymerase was produced by infection with vTF7-3 which contains the T7 RNA polymerase gene linked to a vaccinia virus promoter [65]. The *E. coli lacZ *gene linked to the T7 promoter was introduced into cells by infection with vaccinia virus recombinant vCB21R-LacZ, which was described previously [66]. HeV stock virus (titer 1 × 10^8 ^TCID_50_/ml) was prepared as described [67]. NiV stock virus (titer 3 × 10^7 ^TCID_50_/ml) was prepared as described [68].

### Cell-fusion assays

Fusion between envelope glycoprotein-expressing and target cells was measured by a reporter gene assay in which the cytoplasm of one cell population contained vaccinia virus-encoded T7 RNA polymerase and the cytoplasm of the other contained the *E. coli lacZ *gene linked to the T7 promoter; β-galactosidase (β-Gal) is synthesized only in fused cells [35,69]. Vaccinia virus-encoded proteins were produced by infecting cells (moi = 10) and incubating infected cells at 31°C overnight. Cell-fusion reactions were conducted with the various cell mixtures in 96-well plates at 37°C. Typically, the ratio of envelope glycoprotein-expressing cells to target cells was 1:1 (2 × 10^5 ^total cells per well, 0.2-ml total volume). Cytosine arabinoside (40 μg/ml) was added to the fusion reaction mixture to reduce non-specific β-Gal production [35]. For quantitative analyses, Nonidet P-40 was added (0.5% final) at 2.5 h and aliquots of the lysates were assayed for β-Gal at ambient temperature with the substrate chlorophenol red-D-galactopyranoside (CPRG; Roche Diagnostics Corp.). For inhibition by peptides, serial dilutions of peptides were performed and added to effector cell populations prior to the addition of target cell populations. All assays were performed in duplicate and fusion results were calculated and expressed as rates of β-Gal activity (change in optical density at 570 nm per minute × 1,000).

### Virus infection assay and immunofluorescence

Vero cells were seeded into 96 well plates at 6 × 10^4 ^cells/300 μl and grown to 90% confluence in EMEM-10 at 37°C under a humidified 5% CO2 atmosphere. Peptides were diluted 4-fold in EMEM. Under biohazard level 4 conditions, media were discarded and 100 μl of diluted virus was added to each well and incubated at 37°C for 30 min. Virus dilutions were chosen to generate 50 plaques under these adsorption conditions. Virus inoculum was removed and 200 μl of diluted peptide was added to each well and incubated at 37°C for 18 h. The culture medium was discarded and plates were immersed in ice-cold absolute methanol for at least 20 min prior to air-drying outside the biohazard level 4 facility. Fixed plates were immunolabeled with anti-P monospecific antisera [70]. Briefly, slides were washed in 0.01 M phosphate-buffered saline (PBS), pH 7.2 containing 1% BSA for 5 min. 40 μl of anti-P antiserum (1:200 in PBS-BSA) was applied to each well and incubated at 37°C for 30 min. Slides were rinsed with PBS containing 0.05% Tween 20 (PBS-T) and washed for 5 min in PBS-BSA. 40 μl of FITC labeled goat anti-rabbit antiserum (ICN Pharmaceuticals, Costa Mesa, USA) diluted 1:100 in PBS-BSA was then applied to each well and incubated at 37°C for 30 min. Slides were rinsed again with PBS containing 0.05% Tween 20 (PBS-T) and washed for 5 min in PBS-BSA. Wells were overlaid with glycerol/PBS (1:1) containing DABCO (25 ug/ml) and stored in the dark prior to imaging.

FITC immunofluorescence was visualized using an Olympus IX71 inverted microscope (Olympus Australia, Mt. Waverley, Australia) coupled to an Olympus DP70 high resolution color camera. Image analysis was performed using AnalySIS^® ^image analysis software (Soft Imaging System GmbH, Munster, Germany). Briefly, individual virus syncytia were detected by threshold analysis followed by "hole filling" and subsequently measured to determine the area of each syncytium. To ensure repeatability between images, all procedures were performed as a macro function with fixed parameters. Nine images were analysed for each peptide concentration resulting in the collation of syncytial area data for between 9–36 foci per peptide concentration (average ~15). Measurements were collated and non-linear regression analysis performed using GraphPad Prism software (GraphPad Software, San Diego, CA USA) to determine the IC_50_.

### Peptide synthesis

The following peptide sequence, corresponding to the C-terminal α-helical heptad repeat domain (HR-2) of the NiV F glycoprotein, was chosen for synthesis: KVDISSQISSMNQSLQQSKDYIKEAQRLLDTVNPSL (NiV FC2). A scrambled version of the 36-amino-acid peptide was also synthesized for use as a negative control KQSSMISLQSQKSINSLPSQIRDYVQKTVLLAEDND (ScNiV FC2).

All peptides were synthesized utilizing the Fmoc/tBu protection scheme. The peptides with PEG(_10_) on the N-terminus were synthesized on a PS3 automated synthesizer (Protein Technologies Inc., Tucson, AZ) using NovaSYN^® ^TGR Resin (Nova Biochem, EMD Biosciences, Inc. La Jolla, CA). The peptides with PEG(_10_) on the C-terminus were synthesized on an ABI433 automated synthesizer (Applied Biosystems, Foster City, CA) using 2-Chlorotrityl resin (Nova Biochem). The protected amino acids were incorporated into the peptide via active ester formation using 2-(6-Chloro-1H-benzotriazole-1-yl)-1,1,3,3-tetramethyluronium hexafluorophosphate (HCTU) (Nova Biochem). All Fmoc protected amino acids were supplied by Nova Biochem. The protecting groups used were as follows: sidechains of Asn, Cys, His, and Gln were protected with Trityl (trt), Glu and Ser were protected with *tert*-Butyl (tBu), Lys was protected with *tert*-Butyloxycarbonyl (Boc),Arg was protected with 2,2,4,6,7-pentamethyldihydrobenzopfuran-5-sulfonyl (Pbf). Ala, Leu, Phe, Val, and Gly were used without sidechain protection. PEG_(10) _was incorporated using O-(N-Fmoc-2-aminoethyl)-O'-(2-carboxyethyl)-undecaethyleneglycol (Nova Biochem) and the peptides were acetylated by treatment with acetic anhydride (Sigma-Aldrich). Peptides were cleaved from the solid support using 92% trifluoacetic acid (Halocarbon), 2% anisole, 2% ethanedithiol, 2% triisopropylsilane (all Sigma-Aldrich), and 2% water. Peptides were purified on a Waters 600e semi-prep HPLC system using a grace Vydac 300. Diphenyl column and solvents 0.1%TFA/water (A) and 0.1%TFA/acetonitrile (ACN) (B). Analytical HPLC analysis of all fractions was performed using a Waters Alliance 2695 with a 2.1 × 30 mm Symmetry Shield™ RP18 3.5 m column. Matrix assisted laser desorption/ionization time of flight (MALDI-ToF) mass spec analysis of the crude and pure peptides was performed using an ABI Voyager DE Pro system. Crude peptide from each synthesis and pure peptide was dissolved in 50%ACN/water and spotted with α-Cyano-4-Hydroxycinnimic Acid matrix (Sigma-Aldrich). Positive ions were detected using the linear detector, which is calibrated with Bradykinin and Angiotensin standards.

### Proteomics computational methods

Methods to derive general models of surface glycoproteins have been described previously [43]. Homology modelling of Hendra virus and Nipah virus F was based on the structure of the F protein of Newcastle disease virus, another member of the Paramyxoviridae, determined by x-ray crystallography [40]. MacMolly (Soft Gene GmbH, Berlin) was used to locate areas of sequence similarity and to perform alignments. PHDsec (Columbia University Bioinformatics Center, /) was used for secondary structure prediction [41]. PHDsec predicts secondary structure from multiple sequence alignments by a system of neural networks, and is rated at an expected average accuracy of 72% for three states, helix, strand and loop. Domains with significant propensity to form transmembrane helices were identified with TMpred (ExPASy, Swiss Institute of Bioinformatics, ). TMpred is based on a statistical analysis of TMbase, a database of naturally occurring transmembrane glycoproteins [42].

## Competing interests

The author(s) declare that they have no competing interests.

## Authors' contributions

KNB conceived and contributed to the design and use of heptad derived peptides as fusion inhibitors henipaviruses, designed and carried out all cell-fusion assays, interpreted data, and edited and corrected the manuscript. BAM and GC developed and carried out all live virus infections and peptide inhibition assays, interpreted data and edited and corrected the manuscript. LFW provided financial support, corrected the manuscript and provided supervision of KNB. BTE provided expertise for conducting the live virus infection experiments, financial support, corrected the manuscript and provided supervision of BAM and GC. CCB conceived and contributed to design and use of heptad derived peptides as fusion inhibitors for henipaviruses and PEG-linked versions of peptides, provided overall supervision and financial support and prepared the final versions of the manuscript.
